# Vitamin K supports TGF-β1 depended *in vitro* human Langerhans cell differentiation and function via Axl

**DOI:** 10.3389/fimmu.2025.1509228

**Published:** 2025-02-18

**Authors:** Thomas Bauer, Susanne Richter-Eder, Nighat Yasmin, Jennifer Jurkin, René Köffel, Herbert Strobl

**Affiliations:** ^1^ Center for Cancer Research, Medical University of Vienna, Vienna, Austria; ^2^ Comprehensive Cancer Center, Medical University of Vienna, Vienna, Austria; ^3^ Institute of Immunology, Center of Pathophysiology, Infectiology and Immunology, Medical University of Vienna, Vienna, Austria; ^4^ Division of Immunology, Otto Loewi Research Center, Medical University of Graz, Graz, Austria

**Keywords:** dendritic cell, Langerhans cell, keratinocyte, transforming growth factor, receptor tyrosine kinase Axl

## Abstract

**Introduction:**

On the outermost edge of the body a dense network of dendritic cells (DCs), the so-called Langerhans cells (LCs), represents the first immune barrier. The establishment and maintenance of this epidermal network is dependent on the cytokine transforming growth factor-β1 (TGF-β1) expressed by keratinocytes (KC) and LCs. We recently identified a crucial downstream effector of TGF-β1, the receptor tyrosine kinase Axl. Axl belongs to the TAM receptor family, which also includes Tyro3 and Mer, and is activated through the vitamin K-dependent ligands Gas6 and Protein S.

**Methods:**

We have now established that TGF-β1 dependent *in vitro* human LC generation from CD34^+^ progenitor cells can be enhanced by Axl over-expression.

**Results:**

Additionally, we supplemented vitamin K into serum-free human LC generation cultures in order to activate the endogenous ligands Gas6 and Protein S. Vitamin K exhibited supportive effects on LC differentiation and LC-associated gene expression. The vitamin K antagonist warfarin on the other hand, hindered efficient LC differentiation. Blocking antibodies against Axl abrogated the positive effect of vitamin K on LC differentiation. Lastly, vitamin K downregulated the immune activation marker CD86 during LC differentiation and blocked the upregulation of CD86 during LC activation *in vitro*, in an Axl independent manner.

**Discussion:**

Taken together, we provide evidence for the supportive role of vitamin K in regulating skin immunity.

## Introduction

TGF-β1 is a fundamental immunosuppressive cytokine (1). Mice devoid of TGF-β1 are born normally but develop a devastating inflammatory disease. Although nearly all organs are involved, severe gut inflammation is most prominent, leading to a wasting syndrome and fatality after postnatal week two (2). Along with the inflammation, these mice also develop autoimmunity, with detectable autoantibodies to widespread epitopes (3).

TGF-β1 is expressed in the epidermal microenvironment and is crucial for the development of the LC network (4). TGF-β1 is considered the master regulator of LC differentiation, given that TGF-β1 null mice lack LCs and that TGF-β1 can induce LC differentiation from monocytopoietic precursors and monocytes *in vitro* (5–8). Several transcription factors downstream of TGF-β1 have been identified as necessary for LC differentiation; i.e. Id2 (inhibitor of differentiation and DNA binding 2) and Runx3 (Runt related transcription factor 3) (9, 10).

In addition to its role in mediating LC differentiation, TGF-β1 is crucial for maintaining an intact LC network. Conditional knockout mice (KO), for TGF-β1 or its receptor ALK5 in LCs revealed spontaneous activation and migration of LCs, leading to the complete abolishment of the epidermal LC network (11, 12). Similarly, DC-specific ALK3 KO mice exhibit a perturbed LC network, with ALK3 having anti-inflammatory properties on DCs (13, 14).

Although it has been known for several years that TGF-β1 is important for keeping dendritic cells in an immature state (8), downstream factors or signaling events remained poorly understood.

We recently identified the receptor tyrosine kinase Axl as being specifically expressed downstream of TGF-β1 in LCs (15). Knockout mice lacking Axl and its closely related family members Tyro3 and Mer (TAM family of receptor tyrosine kinases), displayed a pronounced destabilization of the LC network, with some mice almost completely lacking LCs (15). Furthermore, these mice displayed an enhanced response to skin inflammation, indicating the essential immunomodulatory role of LCs, TGF-β1, and Axl (15). Interestingly, the ligands of the TAM receptors, Gas6 and Protein S, are vitamin K-dependent proteins, that require vitamin K for their post-translational carboxylation and, consequently, for their activity as TAM ligands (16).

In this study, we used a human *in vitro* LC generation protocol from CD34^+^ hematopoietic stem cells to investigate the effects of Axl activation and inhibition during TGF-β1 dependent LC differentiation and activation. We found that adding vitamin K to the LC cultures enhanced LC differentiation in an TGF-β1-Axl-dependent manner and that vitamin K can downregulate the activation potential of LCs, implicating its anti-inflammatory potential. This highlights the importance of vitamin K in regulating cutaneous immunity.

## Results

### Axl, Gas6 and Protein-S are expressed by human CD34^+^ derived LCs and Axl over-expression promotes LC differentiation

**Figure 1:**
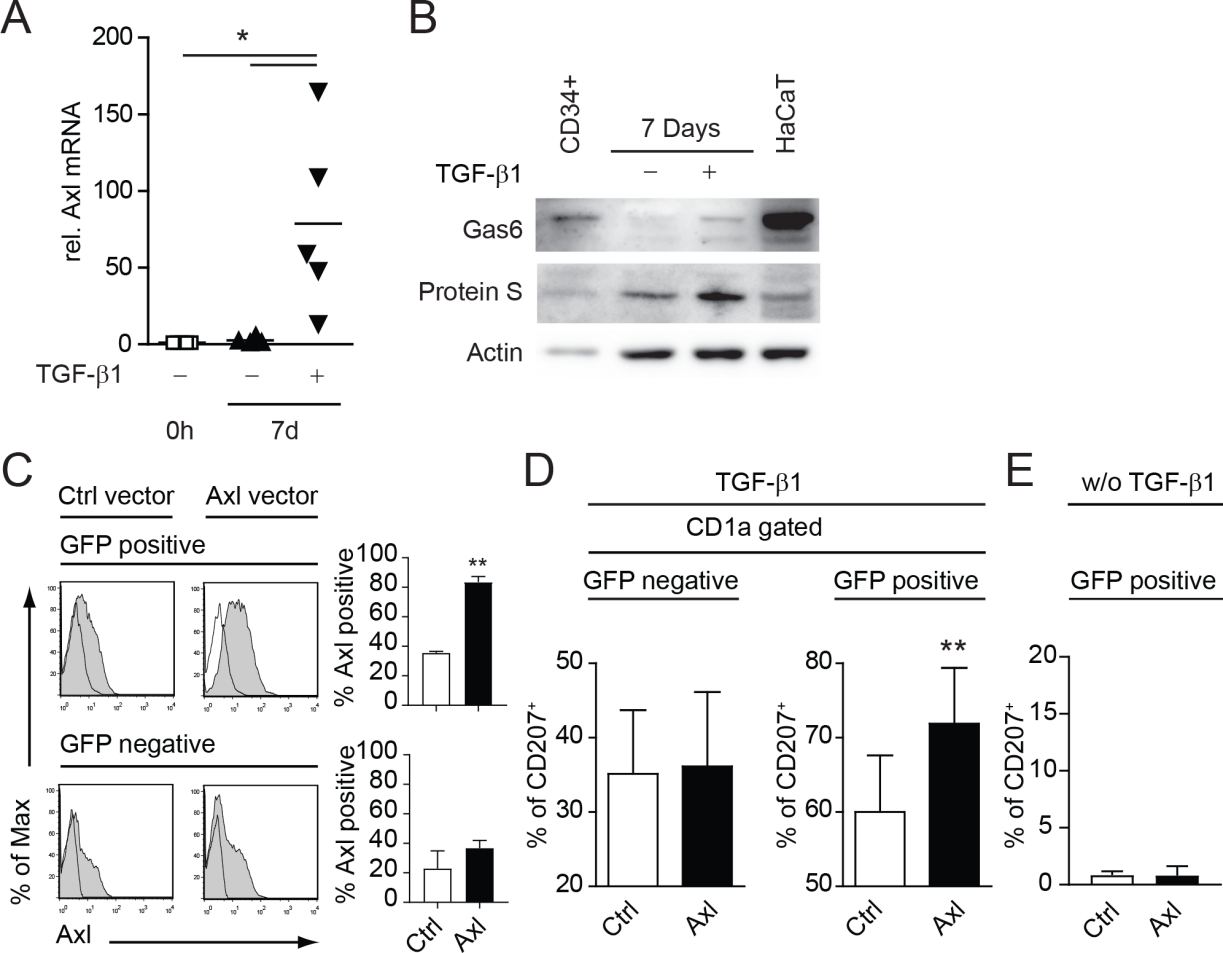
Axl, Gas6 and Protein-S are expressed by human CD34^+^ derived LCs and Axl over-expression promotes LC differentiation *in vitro*. **(A)** CD34^+^ cells were cultured for 7 days (d) in LC promoting conditions (GM-CSF, SCF, Flt-L, TNFα with or without TGF-β1) and Axl mRNA expression was measured relative to 0 h by quantitative real-time RT-PCR. Values were normalized to HPRT. Data represents five different donor experiments. **(B)** CD34^+^ cells were cultured for 7 days in LC promoting conditions (GM-CSF, SCF, Flt-L, TNFα with or without TGF-β1) and analysed for Gas6 and Protein-S protein levels by western blot. HaCaT cells were used as a positive control. Data are representative of three independent donor experiments. **(C)** CD34^+^ cells were transduced with retroviral vectors encoding Axl-IRES-GFP (Axl) or empty control vector (Ctrl) and were cultured in LC promoting conditions as described in **(A, B)**. GFP positive and negative LCs were analysed for Axl surface expression by FACS. Bar diagrams summarize data from more than 3 donor experiments. **(D)** Axl or Ctrl transduced LCs were gated for GFP and CD1a and analysed for CD207 expression by FACS. Bar diagram represents percentages of CD207^+^ cells (± SEM) from more than 3 donor experiments. **(E)** Bar diagram represents data from **(D)** without the addition of TGF-β1 from 2 independent experiments with 2 independent human donors. **P < 0.005. Data in **(A)** are shown as means (± SEM). *P < 0.05 as determined by one-way ANOVA with Tukey´s *post hoc* test.

We could recently show that the TAM receptor Axl is specifically induced during LC differentiation from human umbilical cord blood CD34+ hematopoietic progenitor/stem cells (15). Axl mRNA was directly induced by TGF-β1 in LC progenitor cells (15). In a first step we verified that Axl mRNA levels remain elevated after the 7d differentiation process (**Figure 1A**). Secondly, we investigated the expression of the TAM receptor ligands Gas6 and Protein S. Both ligands were expressed in CD34^+^ cells and in cells cultivated 7 days with LC promoting cytokines (GM-CSF, Flt3L, SCF, TNFα) ± TGF-β1 under serum free conditions (**Figure 1B**). Gas6 was highly expressed in CD34^+^ cells with a marked down-modulation during the differentiation process (**Figure 1B**). However, detectable levels of Gas6 and Protein S were present in TGF-β1 supplemented cultures until the culture-endpoint (**Figure 1B**). Notably, the human KC cell line HaCaT expressed high amounts of Gas6, as also observed in human skin (15). We previously established that Axl expression precedes the classical human LC markers CD1a and CD207 (Langerin), thus pointing to a functional involvement of this receptor tyrosine kinase during the LC differentiation process (15). Using retroviral over-expression vectors harboring Axl and IRES-EGFP, we could further enhance Axl surface expression during LC differentiation (**Figure 1C**). We observed a significant enhancement of CD207^+^ cells from the GFP positive Axl expressing LC fraction as compared to the empty control vector (**Figure 1D**, right bar diagram). GFP negative fractions remained unaltered and served as an internal control (**Figure 1D**, left bar diagram). No CD207 expression was observed in cultures infected with Axl or empty vector control without TGF-β1 addition (**Figure 1E**). Thus, Axl supports TGF-β1 dependent LC differentiation *in vitro*.

### Vitamin K promotes and warfarin impairs *in vitro* LC differentiation and LC numbers

**Figure 2:**
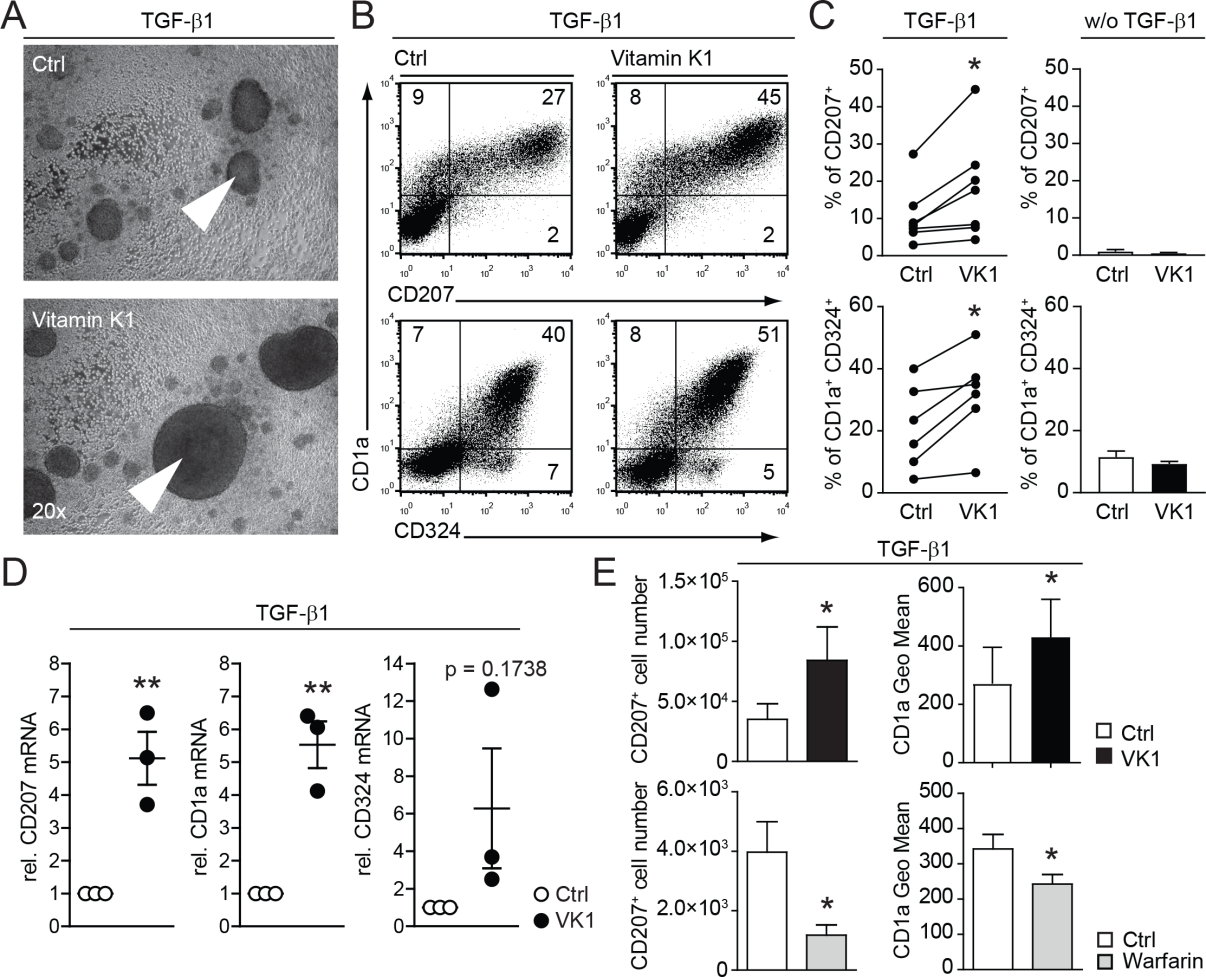
Vitamin K promotes and warfarin impairs *in vitro* LC differentiation and LC numbers. **(A)** CD34^+^ cells were cultured for 7 days in LC promoting conditions (with TGF-β1) with or without vitamin K (Konakion, Roche; 1μg/ml). Cell cluster formation was analysed by bright field microscopy. Pictures are representative of more than three independent donor experiments. Arrowheads indicate representative cell cluster. **(B)** Representative FACS plots from LCs generated with or without addition of vitamin K Cells were analysed for CD1a, CD207 and CD324. **(C)** % of CD207 and CD1a, CD324 positive cells were analysed from **(B)** and depicted as bar graphs (every dot represents an paired independent donor experiment). Cultures without TGF-β1 serve as negative control (right graphs, summarized from 2 independent donor experiments). **(D)** LCs were generated as described in **(A)** and LC clusters were purified by 1g sedimentation. Cell numbers were equalized and mRNA levels for CD1a, CD324 and CD207 were measured by quantitative real-time RT-PCR. Values were normalized to HPRT. LCs generated with vitamin K are depicted relative to control cultures. Dots represent the 3 independent donor experiments performed. **(E)** CD34^+^ cells were cultured for 7 days in LC promoting conditions with and without vitamin K and with or without warfarin (Sigma; 50ug/ml). CD1a surface expression levels and LC numbers were calculated from FACS analysed cells per ml and depicted in the graph. *P < 0.05. Bars represent mean ± SEM.

Taken this positive effect of Axl over-expression on the LC differentiation, we speculated that vitamin K could influence the activity of the endogenous ligands Gas6 and Protein S, which are constitutively expressed in our LC culture system (**Figure 1B**). The functionality of Gas6 and Protein S is regulated by post-translational vitamin K-dependent carboxylation (16). Indeed, addition of vitamin K to human CD34^+^ cell based TGF-β1 dependent *in vitro* LC differentiation cultures resulted in enhanced generation of large LC clusters, an indication for a positive effect on LC generation (**Figure 2A**). Accordingly, FACS analyses revealed increased percentages of CD1a, CD324 (Ecadherin) and CD207 positive (**Figures 2B, C**). This positive effect on LC generation was strictly TGF-β1 dependent, as omitting TGF-β1 in vitamin K cultures did not induce LCs (**Figure 2C** right graphs). The changes of LC specific surface markers (i.e. CD207 and CD1a) are also reflected on the mRNA level as measured by real time RT-PCR (**Figure 2D**). Therefore, vitamin K promotes LC generation *in vitro*.

Warfarin antagonizes the recycling of endogenous vitamin K in the cell, thus inactivating vitamin K-dependent proteins (e.g. blood coagulation factors) (17). Expectedly, the addition of warfarin to LC cultures resulted in the opposite effect of vitamin K supplementation: LC numbers (CD207^+^ cells) were diminished; additionally the surface expression of CD1a was reduced (**Figure 2E**). Thus, vitamin K-dependent processes favor the development of human LCs *in vitro*.

### Vitamin K promotes *in vitro* LC differentiation through Axl

**Figure 3:**
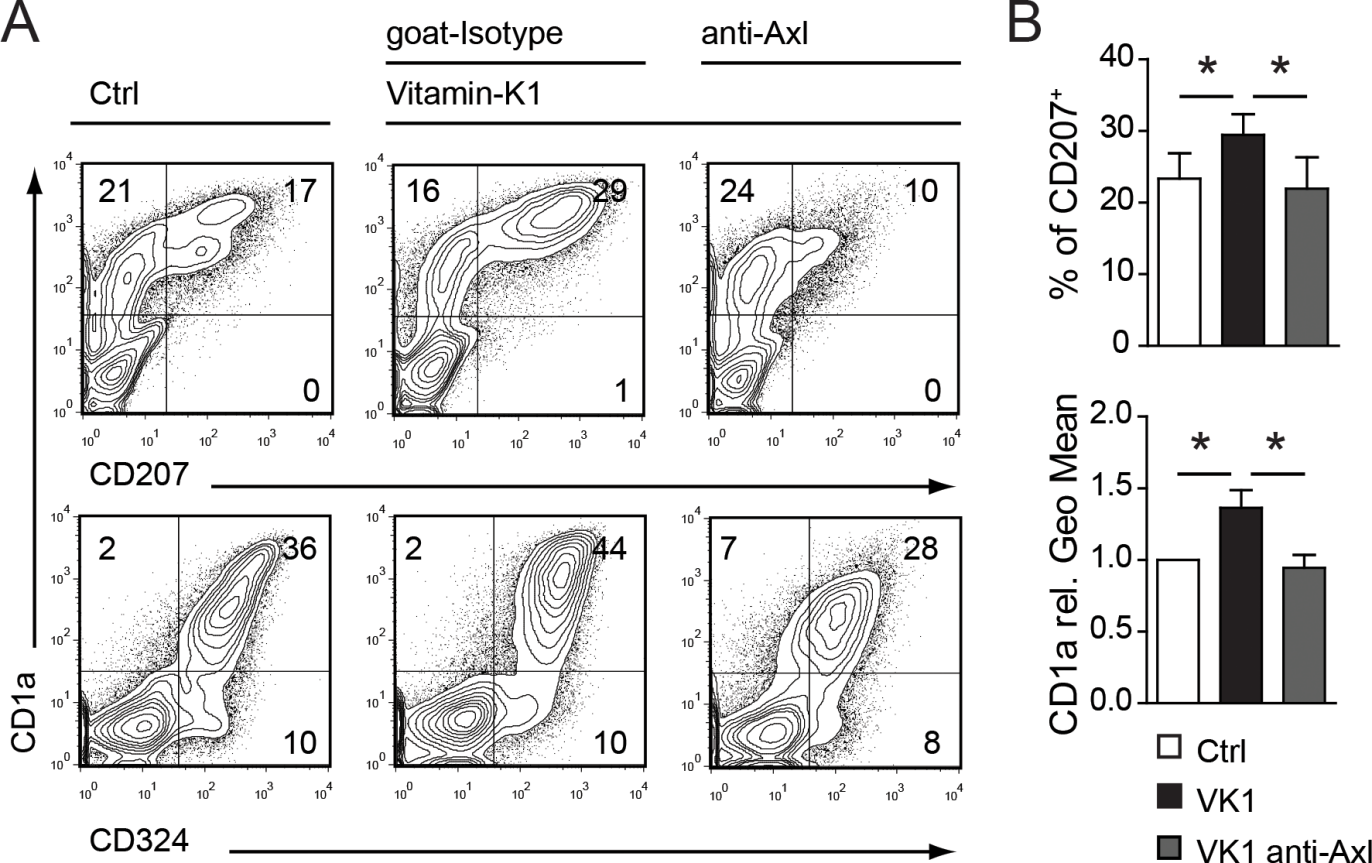
Vitamin K promotes *in vitro* LC differentiation through Axl. **(A)** CD34^+^ cells were cultured for 7 days in LC promoting conditions with or without vitamin K (Konakion, 1μg/ml). Isotype control or anti-Axl Ab (R&D systems; AF154) was added as depicted (5 μg/ml on d0 and d3). LCs were analysed for the indicated surface receptors by FACS. Data are representative of more than three independent donor experiments. **(B)** Cells from **(A)** were analysed by FACS. Bars represent mean ± SEM. *P < 0.05.

Given the positive effect of vitamin K on LC generation and the here identified expression of the vitamin K-dependent receptor-ligand system Axl-Gas6-Protein S during LC differentiation (**Figures 1**, **2**), we next investigated its direct involvement. We used a blocking antibody to silence Axl during human *in vitro* LC differentiation (15, 18). Antibodies against Axl, administered during the *in vitro* differentiation, efficiently blocked the positive effect of vitamin K on LC generation (**Figures 3A, B**). While warfarin reduced CD1a surface expression, vitamin K enhanced the levels of CD1a on LCs in an Axl dependent manner (compare **Figures 2E**, **3B**, lower graph). Thus, TGF-β1 promotes *in vitro* LC differentiation through the early up-regulation of Axl, which is activated by its endogenous vitamin K-dependent ligands in these cultures.

### Vitamin K impairs LC activation by TLR ligands and cytokines

**Figure 4:**
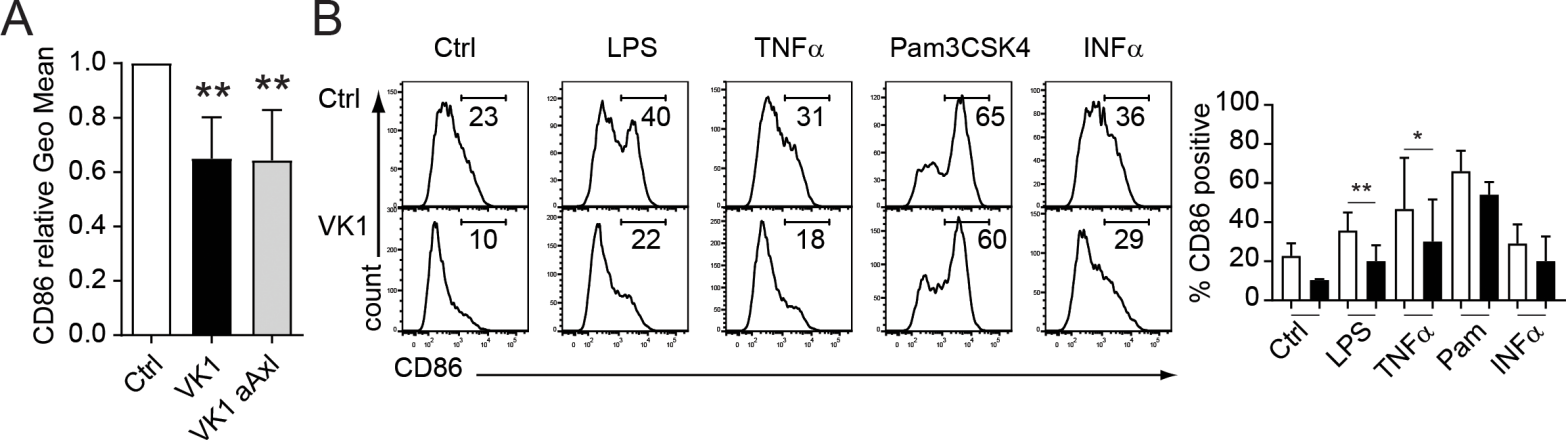
Vitamin K impairs LC activation by TLR ligands and cytokines. **(A)** CD34^+^ cells were induced to differentiate into LCs in the presence (dark bar) or absence (empty bar) of vitamin K (Konakion, 1μg/ml) and anti-Axl blocking Abs (grey bar). Day 7 generated CD1a^+^ CD207^+^ LCs were analysed for CD86 by FACS. **(B)** LCs were generated as described in **(A)**. Vitamin K was added 3 h prior activation with the indicated stimuli. Cells were harvested 24h after stimulation and analysed for CD86 expression by FACS. Data are representative of at least three independent donor experiments summarizes as bar graphs (right side). *P < 0.05, **P < 0.01. Bars represent mean ± SEM.

Both Axl and its ligands (Gas6 and Protein-S) are implicated to have anti-inflammatory effects (19). We therefore investigated the impact of vitamin K on co-stimulatory molecule expression by *in vitro* generated LCs. Whereas vitamin K enhanced the expression of CD207 and CD1a (**Figure 2**), the surface levels of the costimulatory molecule CD86, which is necessary for T-cell activation and survival, were significantly diminished (**Figure 4A**). Interestingly, this effect was Axl independent, suggesting a broader anti-inflammatory role of vitamin K in LCs (**Figure 4A**). Furthermore, the addition of vitamin K to LCs prior to their stimulation with TLR-ligands (e.g. LPS) or cytokines (e.g. TNFα) led to a blunted up-regulation of CD86 (**Figure 4B**).

**Figure 5:**
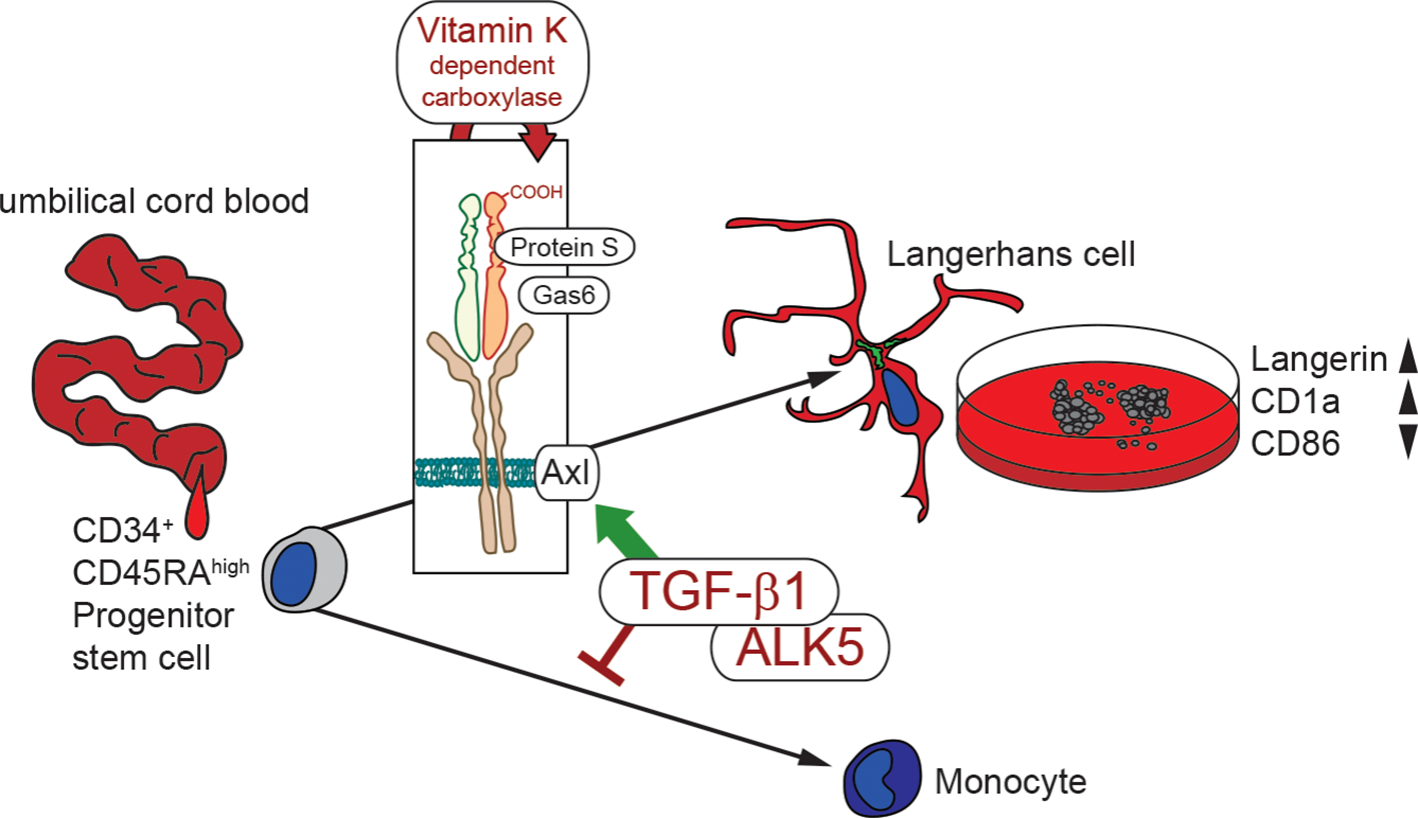
Schematic illustration of *in vitro* LC generation in the presence of vitamin K. Hematopoietic stem cell from umbilical cord blood are instructed by TGF-β1 for LC commitment. TGF-β1 additionally up-regulates Axl, which is activated by Gas6 and Protein S. Vitamin K dependent carboxylation facilitates ligand activity, thereby enhancing LC generation (high Langerin and CD1a) with lower immune activation potential (low CD86).

In summary, these data indicate that vitamin K enhances LC differentiation, through Axl activation downstream of TGF-β1 signaling, while it simultaneously counteracts LC activation (**Figure 5**).

## Discussion

This study implicates vitamin K as a supportive factor for skin immunity. The vitamin K-dependent proteins Gas6 and Protein S drive cutaneous TGF-β1-dependent LC generation *in vitro* through Axl, while vitamin K is simultaneously dampening their co-stimulatory properties. This suggests an additional anti-inflammatory role of vitamin K in the skin.

TGF-β1 is a fundamental regulatory cytokine of the immune system. It negatively regulates inflammation and positively controls immune cell development (1). TGF-β1 is especially important for barrier immunity. TGF-β1 KO mice exhibit severe gut inflammation that develops after birth, leading to a lethal wasting syndrome (2). In the lung, TGF-β1 is produced by macrophages after apoptotic cell encounter following inflammation, which is crucial for its resolution (20). The immune system of the skin´s epidermal layer, the LC network, is critically dependent on TGF-β1 expression in the stratified epithelium (5).

Downstream of TGF-β1 and its main receptor, ALK5, Axl is a key effector that is regulated by its ligands Gas6 and Protein S (15). This enabled us to modulate their activation by manipulating the vitamin K levels in the LC cultures. Interestingly, Gas6, similar to TGF-β1, is expressed in the upper KC layers in human skin (15). Addition of vitamin K enhances ligand activity, while of warfarin blocks vitamin K dependent processes. The here described culture method, using vitamin K-supplemented media, thus represents an improvement in human LC differentiation *in vitro* and may be applicable for DC-based cell therapy, where the generation of large numbers of DCs is of interest.

LCs represent the first layer of immune cells at the outermost part of the body and are the first to be immunologically activated upon skin and hair barrier insults (21–23). The regulation of Axl is primarily associated with inflammation, with Axl being strongly induced following the inflammatory activation of DCs and macrophages (19, 24). This upregulation blocks pro-inflammatory cytokine production in the context of a negative feedback loop to prevent immune over-reactions and autoimmunity (19).

Vitamin K has been implicated in having anti-inflammatory properties in the context of various systemic infections (e.g. coronavirus-induced disease) and autoimmune diseases (e.g. multiple sclerosis and rheumatoid arthritis) (25). The constitutively active vitamin K–Axl axis on LCs may, therefore, render these cells more resilient to inflammatory and stress stimuli, thereby preventing unnecessary immune reactions at the skin barrier. Boosting this system by topically applying vitamin K to the skin could be useful for down-modulating unnecessary immune reactions in certain skin diseases.

Taken together, this study implicates vitamin K in supporting skin immune development, maintenance, and function.

## Materials and methods

### Isolation of primary human cells

Cord blood samples from healthy donors were collected during healthy full-term deliveries. CD34^+^ cells were isolated as described (26). CD14^+^ monocytes were isolated from peripheral blood of healthy donors as described (26). All procedures were carried out in accordance to the guidelines from the Medical University of Vienna Institutional Review Board for these studies. Informed consent was provided in accordance with the Declaration of Helsinki Principles.

### Cytokines and reagents

Human stem-cell factor (SCF), thrombopoietin (TPO), tumor necrosis factor alpha (TNFα), granulocyte-macrophage colony-stimulating factor (GM-CSF), and fms-related tyrosine kinase 3 ligand (FLT3L) were obtained from PeproTech; transforming growth factor beta 1 (TGF-β1), interferon-alpha (IFN-α), mouse GM-CSF from Akron Biotech. Ultrapure LPS from *Escherichia coli* and Pam_3_CSK_4_ was purchased from InvivoGen.

### *In vitro* culture of primary human cells

CD34^+^ cord blood cells were cultured serum-free for 2–3 days under progenitor expansion conditions (Flt3L, SCF and TPO, each at 50 ng/ml) before sub-culturing with lineage-specific cytokines. LC cultures were described before (27). Briefly, CD34^+^ cells (5 × 10^4^ to 1 × 10^5^/ml per well) were cultured in 24-well tissue culture plates in serum-free CellGro DC medium (CellGenix) supplemented with GM-CSF (100 ng/ml), SCF (20 ng/ml), Flt3 (50 ng/ml), TNF-α (2.5 ng/ml) and TGF-β1 (1 ng/ml) for 7 days. Cultures were supplemented with GlutaMAX (2.5 mM; Gibco/Invitrogen) and penicillin/streptomycin (125 U/mL each). Vitamin K (Konakion, Roche) was added at 1μg/ml as indicated. Warfarin (Sigma) was added at 50 μg/ml when indicated. Axl blocking antibody from R&D systems (AF154) was used at a concentration of 5 μg/ml when indicated.

### Retroviral vectors, transfection of packaging cell lines, and gene transduction

The AXL-IRES-GFP retroviral vector was a kind gift from Axel Ullrich (Max Planck Institute of Biochemistry, Martinsried, Germany). Transfection of packaging cell line phoenix-GP as well as infection of target cells was performed as previously described (28). In brief, to produce recombinant amphotropic retrovirus, vectors were transiently transfected into the packaging cell line Phoenix-GP (Gag-Pol) using a calcium-phosphate protocol (28). Phoenix-GP was cotransfected with an expression plasmid encoding gibbon ape leukemia virus (GALV) envelope (gift from D.B. Kohn, University of California, Los Angeles, Los Angeles, CA). Before gene transduction, fresh or thawed CD34^+^ cells were stimulated overnight in X-VIVO 15 medium supplemented with the cytokines SCF (50 ng/ml), FLT3L (50 ng/ml), and TPO (50 ng/ml). Afterwards, 1 ml of retroviral supernatant (harvested 36–48 h after transfection of packaging cells) was added to 4 × 10^4^ CD34^+^ HPCs in the presence of plate-bound RetroNectin (Takara Bio Inc.) using nontissue culture–treated 24-well plates (Cellstar; Greiner Bio-One GmbH) according to the instructions of the manufacturer. Infections were repeated two to three times at intervals of 12–24 h using fresh virus supernatants in the presence of cytokines SCF, FLT3L, and TPO. Within 60 h of the first transduction cycle, cells were harvested and re-cultured in LC lineage conditions.

### RNA isolation and real time RT-PCR

RNA was isolated using RNeasy Mini Kit (Qiagen). 1 μg of RNA was reverse transcribed using Transcriptor First Strand cDNA Kit (Roche) and oligo(dT) primers. Quantitative PCR was run on Roche LightCycler (human samples) using the SYBR Green PCR Master Mix (Applied Biosystems) and indicated primers at 125 nM concentration. Results were analyzed using delta delta Ct method and presented as a fold of difference in mRNA level relative to HPRT (human samples) (29).

### Flow cytometry

Flow cytometry staining and analysis were performed as described (28). Monoclonal antibodies (mAbs) of the following specificities were used: FITC-conjugated mAbs specific for CD1a, CD86 (BD Biosciences), Phycoerythrin (PE)-conjugated mAbs specific for CD207 (Immunotech), Allophycocyanin-conjugated Abs against CD1a (BD Biosciences) and CD324 (Biolegend); Pacific Blue conjugated mAbs against CD1a (Biolegend); biotinylated mAbs specific for CD86 (BD Biosciences). Second step reagent was streptavidin (SA)-PerCP (BD Biosciences). Axl was detected using mouse mAbs (R&D systems) followed with a PE conjugated anti mouse second step Ab (Dako). Flow cytometric analysis was performed using a LSRII instrument (BD Biosciences) and the FloJo software (Tree Star, Inc.).

### Western blot analysis

Cells were lysed in lysis buffer containing 50 mM Tris-HCl pH 7.5, 1 mM EGTA, 1 mM EDTA, 1% (w/v) Triton X-100, 0.27 M sucrose, 0.1% 2-mercaptoethanol, protease inhibitor cocktail (Roche) and phosphatase inhibitor cocktail (Roche). Protein concentration was measured using Bradford reagent (Bio-Rad) and equal amount of protein (5 μg) in LDS sample buffer (Invitrogen) were subjected to electrophoresis on a polyacrylamide gel and transferred to PVDF membranes (Millipore). Membranes were blocked in TBS-T (50 mM Tris-HCl pH 7.5, 0.15M NaCl, and 0.25% (v/v) Tween-20) containing in 5% (w/v) BSA. The membranes were then immunoblotted overnight at 4°C with primary antibodies diluted 1000-fold in blocking buffer. The blots were washed six times with TBS-T and incubated for 1 h at room temperature with secondary HRP-conjugated antibodies diluted 5000-fold in 5% (w/v) skimmed milk in TBS-T. After repeating the washing steps, signal was detected with the enhanced chemiluminescence reagent and immunoblots were developed using an automatic film processor.

### Statistical analysis

If not specified in figure legends, statistical analysis was performed using the paired or unpaired, 2-tailed Student t test; *p-*values of less than 0.05 were considered significant.

## Data Availability

The raw data supporting the conclusions of this article will be made available by the authors, without undue reservation.
