# Supplementary figures and images for: Vitamin K supports TGF-β1 depended in vitro human Langerhans cell differentiation and function via Axl
Source: Front Immunol. 2025 Feb 18;16:1509228. doi: 10.3389/fimmu.2025.1509228 (PMC11876179; doi:10.3389/fimmu.2025.1509228)

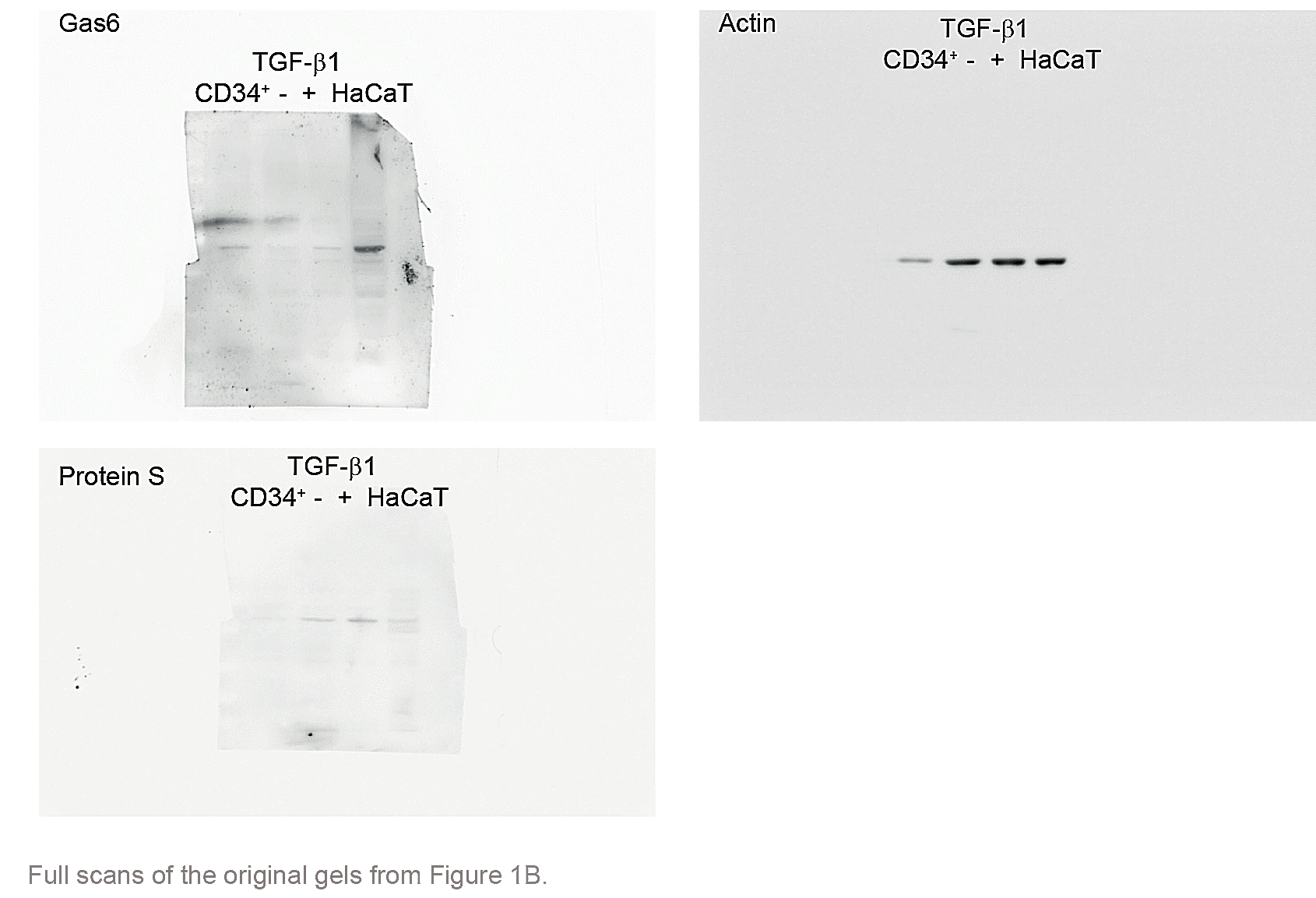

Supplement: Supplementary file 1 [file Image1.tif]
